# ER–Mitochondria Contacts and Insulin Resistance Modulation through Exercise Intervention

**DOI:** 10.3390/ijms21249587

**Published:** 2020-12-16

**Authors:** Yi Sun, Shuzhe Ding

**Affiliations:** 1 Key Laboratory of Adolescent Health Assessment and Exercise Intervention of Ministry of Education, East China Normal University, Shanghai 200241, China; ysun@tyxx.ecnu.edu.cn; 2 College of Physical Education and Health, East China Normal University, Shanghai 200241, China

**Keywords:** MAMs, insulin resistance, exercise, obesity, diabetes

## Abstract

The endoplasmic reticulum (ER) makes physical contacts with mitochondria at specific sites, and the hubs between the two organelles are called mitochondria-associated ER membranes (MAMs). MAMs are known to play key roles in biological processes, such as intracellular Ca^2+^ regulation, lipid trafficking, and metabolism, as well as cell death, etc. Studies demonstrated that dysregulation of MAMs significantly contributed to insulin resistance. Alterations of MAMs’ juxtaposition and integrity, impaired expressions of insulin signaling molecules, disruption of Ca^2+^ homeostasis, and compromised metabolic flexibility are all actively involved in the above processes. In addition, exercise training is considered as an effective stimulus to ameliorate insulin resistance. Although the underlying mechanisms for exercise-induced improvement in insulin resistance are not fully understood, MAMs may be critical for the beneficial effects of exercise.

## 1. Introduction

Recent studies have shown that cellular organelles perform proper functions by interacting with other organelles. Mitochondria are dynamic organelles that play a key role in energy conversion. Mitochondria exhibit structural and functional diversity, and subsets of mitochondria form physical contacts with other types of organelles, including the endoplasmic reticulum (ER) [1], peroxisomes [2], lipid droplets [3], etc. The ER is the main site for the synthesis of proteins and lipids, membrane biogenesis, xenobiotic detoxification, and cellular Ca^2+^ storage. Proper functions of the mitochondria and ER are essential for maintaining cellular homeostasis. In addition to playing distinct cellular roles, the mitochondria and ER form physical interactions at specific sites, which are called mitochondria-associated ER membranes (MAMs). This unique structure serves as the platform for communication between the two organelles, and is critical for Ca^2+^ crosstalk, lipid transport, energy metabolism, ER redox control, mitochondria dynamics, cell survival, inflammation, etc. [4]. 

Accumulating evidence has suggested that alterations of the structure and function of MAMs are closely associated with numerous diseases, such as diabetes mellitus [5], metabolic syndrome [6], nonalcoholic fatty liver disease (NAFLD) [7], cancer [8], and neurodegenerative diseases [9,10]. However, the specific role of MAMs in insulin resistance is still elusive. Moreover, it remains unclear whether the alleviation of insulin resistance by exercise intervention involves the participation of MAMs. Therefore, the aim of this review is to summarize the current understanding of the structure and functions of MAMs, the association between dysfunctional MAMs and insulin resistance, and the current progress on exercise interventions for MAMs modification and insulin resistance improvement.

## 2. The Structure of MAMs

**Figure 1:**
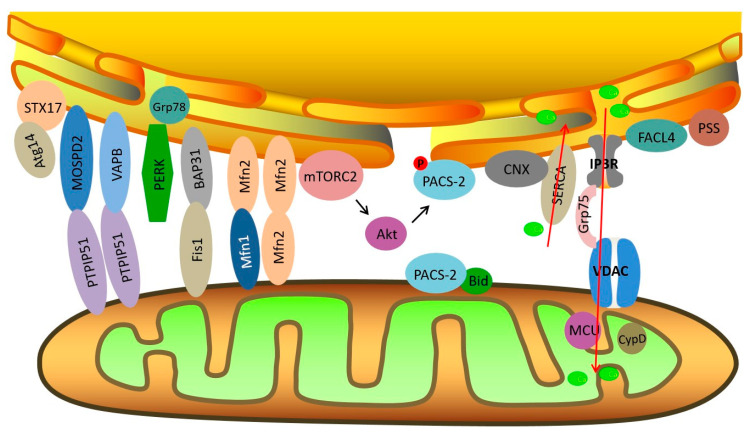
Schematic diagram of endoplasmic reticulum (ER)–mitochondria contact sites and key molecules related to insulin signaling at mitochondria-associated ER membranes (MAMs). Red arrows signify the direction of Ca^2+^ transport.

The physical interactions between the mitochondria and ER depend on complementary membrane proteins that tether the two organelles at specific sites. Several crucial proteins act as tethers between the mitochondria and ER, including PACS-2 (phosphofurin acidic cluster sorting protein 2) [11], Mfn2 (mitofusin 2)-Mfn1/2 [12], IP3R (inositol 1, 4, 5-triphosphate receptor)-Grp75 (glucose regulated protein 75)-VDAC (voltage-dependent anion channel) complex, Fis1-Bap31 [13], VAPB (vesicle-associated membrane-protein-associated protein B)-PTPIP51 (protein tyrosine phosphatase-interacting protein 51) [14], and MOSPD2-PTPIP51 [15] (Figure 1). Some other regulatory proteins are also enriched at MAMs, including calnexin (CNX) [16], sarco/ER Ca^2+^ ATPase (SERCA), sigma-1 receptor (Sig1R) [17], endoplasmic reticulum oxidoreductin-1α (Ero1α) [18], ACSL4/FACL4 [19], phosphatidylserine synthase (PSS) [20], Atg14 [21], CypD (cyclophilin D) [22], Akt [23], mTORC2 (mammalian TOR complex 2), etc. Mitochondria are closely associated with both rough ER and smooth ER. Tethers connecting the OMM (outer mitochondrial membrane) and smooth ER have measured lengths of 9–16 nm, and are measured at 19–30 nm between the OMM and rough ER [24]. In addition, the interaction between mitochondria and ER accounts for about 20% of the total mitochondrial network [25]. In mammalian skeletal muscle, tight coupling is observed between the sarcoplasmic reticulum (SR) and mitochondria. The close interaction between the SR and mitochondria forms bidirectional signaling between the two organelles, which are called “orthograde” (SR to mitochondria) communication and “retrograde” (mitochondria to SR) communication [26]. Ca^2+^ signaling, metabolic processes, muscle fatigue, and muscle damage during exercise are all impacted by SR–mitochondria coupling.

The key molecules and tethers located at MAMs that are associated with insulin sensitivity maintenance are briefly reviewed below.

### 2.1. The IP3R-Grp75-VDAC Complex

Ca^2+^ is an essential second messenger. Intracellular Ca^2+^ homeostasis is maintained partly through Ca^2+^ transfer from the ER to the mitochondria. The VDAC of the outer mitochondrial membrane interacts with IP3R on the ER through the molecular chaperone Grp75, which is a cytosolic Hsp70. This complex allows mouth-to-mouth Ca^2+^ transfer from the ER to the mitochondria [27]. Evidence suggested that deficiency in Grp75 caused a decrease in ER–mitochondria Ca^2+^ exchange [28]. However, excessive entrance of Ca^2+^ into mitochondria would lead to mitochondrial Ca^2+^ overload, triggering apoptosis.

### 2.2. Mfn2

Mfn2, a ubiquitously expressed mitochondrial transmembrane dynamin-like GTPase protein, plays a fundamental role in mitochondrial fusion. Mfn2 was discovered as a direct ER–mitochondria tether, regulating the interactions as well as Ca^2+^ transfer between ER and mitochondria [29]. Ablation of Mfn2 caused fragmentation of the ER, impaired mitochondrial Ca^2+^ uptake, and increased distance between the two organelles [29]. Several molecules, such as trichoplein/mitostatin (TpMs) [30] and MITOL [31], impact ER–mitochondria tethering via interacting with Mfn2.

### 2.3. CypD

CypD is a member of the cyclophilin family, and is located in the mitochondrial matrix. It is important for regulating mitochondrial function and controlling the mitochondrial permeability transition pore (mPTP). To date, the exact role of CypD in regulating mitochondrial function still remains elusive. It is suggested that CypD interacts with the IP3R-Grp75-VDAC1 Ca^2+^-channeling complex at the MAMs’ interface [22]. CypD is crucial for maintaining MAMs integrity through an unknown mechanism, revealed by the fact that significant reduction of MAMs amount was observed in the liver of *CypD-KO* mice [23].

### 2.4. PACS-2

PACS-2 is expressed in the skeletal muscles, heart, brain, and liver, and is involved in various cellular processes, such as membrane trafficking, apoptosis, and autophagy [11]. Confocal microscopy confirmed that PACS-2 was located at MAMs [32]. PACS-2 is essential for the maintenance of MAMs integrity. Depletion of PACS-2 caused mitochondria fragmentation, which uncoupled from the ER [32]. MAMs integrity was also affected by phosphorylation of PACS-2 mediated by mTORC2-Akt signaling [33].

## 3. The Main Functions of MAMs

### 3.1. Lipid Exchange

Most lipids are synthesized in the ER and transported to mitochondria. Therefore, MAMs are crucial for lipid synthesis and trafficking. MAMs contain numerous phospholipid- and glycosphingolipid-synthesizing enzymes, and support the direct transfer of phospholipids from the ER to mitochondria [34].

FACL4 and PSS-1 are both MAMs-localized proteins. FACL4 is the enzyme that converts fatty acids into fatty acyl-CoA esters, and PSS-1 catalyzes the reaction where the polar head group of PE (phosphatidylethanolamine) or PC (phosphatidylcholine) is replaced by L-serine. Knockdown of either PACS-2 or Mfn2 caused a reduced level of MAMs-associated FACL4 and PSS-1 [32,35]. 

### 3.2. Ca^2+^ Homeostasis

Ca^2+^ functions as an important second messenger, and plays a key role in regulating various protein kinases and transcription factors, influencing their expressions or activities. Disruption of Ca^2+^ homeostasis is closely related to several pathologies and chronic diseases, such as cardiac diseases, Alzheimer’s disease, diabetes, etc. [36]. The majority of Ca^2+^ transportation between mitochondria and ER is performed through MAMs. Mitochondria take up Ca^2+^ following an increase in cytosolic Ca^2+^. In addition to modulating cytosolic Ca^2+^ transient, mitochondrial uptake of Ca^2+^ activates key enzymes of the Krebs cycle, thereby regulating mitochondrial ATP production. Therefore, MAMs effectively integrate Ca^2+^ flux with cellular metabolic pathways. However, excessive Ca^2+^ entrance into mitochondria induces the opening of mPTP and release of pro-apoptotic factors, causing detrimental effects on cell viability [37].

The Ca^2+^ level in the cytosol is in the nanomolar range, compared to the millimolar range in the ER [38]. The huge discrepancy is generated by both SERCA, which pumps Ca^2+^ into the ER, and by plasma membrane Ca^2+^ ATPases (PMCA), which pump Ca^2+^ out of the cell [28]. Ca^2+^ transfer from the ER to mitochondria is regulated by the IP3R-Grp75-VDAC1-MCU axis [39]. When ryanodine receptor (RyR) and IP3R—both of which are ER Ca^2+^ channels—open, a transient increase in cytosolic Ca^2+^ level occurs, activating downstream Ca^2+^-binding proteins. Mitochondrial Ca^2+^ uniporter (MCU) is the major regulator of mitochondrial Ca^2+^ uptake. Microdomains of high [Ca^2+^] are created at the MAMs to facilitate rapid Ca^2+^ uptake via the MCU complex. 

The Ca^2+^ crosstalk between the ER and mitochondria is regulated by several factors. For example, CNX interacts with SERCA to regulate intracellular Ca^2+^ oscillations [40]. In addition, PACS-2 is required for oxidized LDL-induced mitochondrial Ca^2+^ elevation, cell apoptosis, and MAMs formation [41]. In addition, knockdown of polycystin 2 (PC2) increased the expression of Mfn2, enhanced the tethering between mitochondria and ER, and caused an increase in mitochondrial Ca^2+^ entry [42]. 

Ca^2+^ also plays an essential role in the regulation of cell death. When mitochondria and ER are aberrantly closely apposed, the mitochondria matrix is overloaded by Ca^2+^, causing the opening of mPTP and release of cytochrome c as well as other apoptotic stimuli [43]. 

### 3.3. Apoptosis

Mitochondrial Ca^2+^ overload is a trigger to stimulate apoptosis. Therefore, prolonged and over-tightened connections between the ER and mitochondria can be converted into apoptotic stimuli [1]. On the other hand, Fis1 and Bap31 bridge mitochondria and the ER by physically interacting with each other. The tripartite protein complex composed of procaspase-8, Bap31, and Fis1 transmits an apoptotic signal from mitochondria to the ER. The interesting part is that instead of directly inducing mitochondrial fission, Fis1 actually interacts with Bap31 to modulate the release of Ca^2+^ from the ER, which then affects mitochondrial fission and apoptosis [13]. 

### 3.4. Autophagy

Macroautophagy is a conserved process by which cells engulf cytoplasmic contents and organelles and fuse with lysosomes for degradation [1]. The origin of the isolation membrane for formation of autophagosomes has been under debate for years. Recent studies suggested that MAMs were the key platform for autophagosome formation [35]. As a pre-autophagosome/autophagosome marker, Atg14 relocated exclusively to MAMs upon starvation [35]. In addition, as the marker of autophagosome formation, the majority of Atg5 was observed at the ER–mitochondria contact site during the entire autophagosome formation process [35]. On the other hand, disruption of MAMs structure also interfered with the autophagic process, revealed by the observation that knockdown of PACS-2 or Mfn2 caused a decrease in Atg14 and LC3II [35]. However, there was also opposing evidence that suggested that tightening the ER–mitochondria connection might actually impair autophagy. For example, overexpression of VAPB (ER protein) and RMDN3 (protein on the outer mitochondrial membrane) tightened ER–mitochondria contacts and inhibited chemical-induced autophagosome formation [44]. Similarly, strengthening VAPB-PTPIP51 tethers to tighten ER–mitochondria contacts also impaired autophagosome formation [14]. Further research suggested that MAMs-mediated autophagy impairment involved enhanced Ca^2+^ uptake by mitochondria, and could be rescued by blocking Ca^2+^ exchange between the ER and mitochondria [14,44].

## 4. Dysfunction of MAMs and Insulin Resistance

Insulin resistance is the state in which responses of peripheral target tissues, including skeletal muscle, adipose tissue, and liver, to the physiological level of insulin are reduced [45]. Since the mitochondria and ER are both essential for maintaining metabolic homeostasis, it is not surprising that impaired functions of the mitochondria, ER, and MAMs are associated with metabolic disturbances such as obesity, insulin resistance, metabolic syndrome, etc. Among the above metabolic disturbances, obesity is closely related to the development of insulin resistance, while insulin resistance plays an essential role during the initiation and progression of metabolic syndrome [11].

### 4.1. MAMs and Insulin Signaling

#### 4.1.1. MAMs Juxtaposition and Integrity

Several imaging techniques have been used to assess the interactions of the mitochondria and ER, including transmission electronic microscopy, electron tomography [46], immunocolocalization of mitochondria- and ER- specific fluorescent proteins [25], and in situ proximity ligation assay (PLA) [23]. Of the above-mentioned techniques, in situ PLA allows for visualization and quantification of interactions between VDAC-IP3R or Grp75-IP3R [47,48]. However, to date, there is no consensus of whether organelles’ juxtaposition is increased or decreased in models of insulin resistance. Some studies reported that obesity caused an abnormal increase in MAMs formation, which resulted in an increased Ca^2+^ flux from the ER to mitochondria in the liver of mice [49]. Others found that a reduction of ER–mitochondria interactions was associated with metabolic alterations in the liver of both *ob/ob* and diet-induced insulin-resistant mice [23]. Insulin resistance in the skeletal muscle has also been proved to be related to disruption of MAMs. A marked decrease in SR/ER–mitochondria interactions (also called MAMs for convenience) and mitochondrial dysfunction were observed in the skeletal muscle of diet-induced obese mice [50]. In addition, MAMs juxtaposition is also affected by nutritional state. ER–mitochondria proximity, which was examined by transmission electron microscopy (TEM) analysis, was reduced in the liver of fed mice compared with fasted mice [51].

Some evidence has suggested that MAMs integrity is required for maintaining insulin sensitivity. For example, it has been found that genetic or pharmacological inhibition of CypD disrupted MAMs integrity and impaired insulin signaling in primary hepatocytes [23]. Conversely, overexpression of CypD increased organelle contacts and enhanced insulin action in primary hepatocytes of diabetic mice. In addition, treatment of *CypD KO* mice with metformin improved both MAMs integrity and insulin sensitivity [23]. A similar role of Mfn2 in maintaining MAMs integrity and insulin sensitivity was also observed. For example, liver-specific Mfn2 KO mice showed glucose intolerance, enhanced hepatic gluconeogenesis, and impaired response to insulin [12]. Conversely, overexpression of Mfn2 improved high-fat diet (HFD)-induced hepatic insulin resistance in rats [52]. Moreover, ER–mitochondria contacts in POMC neurons of the hypothalamus were decreased in diet-induced obesity. Deletion of Mfn2 in POMC neurons disrupted proper mitochondrial–ER homeostasis, and led to reduced energy expenditure, leptin resistance, and obesity [53]. The impact of MAMs integrity maintenance on insulin sensitivity in the adipocytes has also been investigated. Cisd2 is a causative gene associated with Wolfram syndrome. Cisd2 and GTPase of immune-associated protein 5 (Giamp5) were found to interact with each other at MAMs and to regulate intracellular Ca^2+^ homeostasis by promoting mitochondrial Ca^2+^ uptake [54]. It was noticed that a deficiency in Cisd2 caused elevation in the cytosolic Ca^2+^ level, activating Ca^2+^-calcineurin-dependent pathway and downregulating genes related to mitochondrial biogenesis. Therefore, mitochondrial dysfunction and impaired insulin sensitivity in adipocytes were observed [54]. Even though the above evidence supported the positive role of MAMs integrity in maintaining systemic and tissue insulin sensitivity, opposite observations were also seen with regard to PACS-2. A study demonstrated that knockdown of PACS-2, the protein essential for ER–mitochondria tethering, improved insulin sensitivity in the obese mice [49]. Therefore, it seems that proper MAMs juxtaposition and integrity are required in order to maintain insulin sensitivity. ER–mitochondria contact that is tighter than normal would cause mitochondrial Ca^2+^ overload, trigger cellular apoptosis, and inhibit autophagy. Conversely, loss of ER–mitochondria tethers impairs normal exchange of lipids and Ca^2+^ between the ER and mitochondria, thus disturbing hepatic and muscle insulin action [48].

#### 4.1.2. Insulin Signaling Molecules at MAMs

In the normal conditions, the action of insulin is achieved by interacting with insulin receptors (IRs) on the cell surface. The binding of insulin to the α subunit of an IR causes activation of the β subunit and, therefore, tyrosine-phosphorylating insulin receptor substrates (IRSs). IRSs then interact with and activate PI3K, causing translocation of GLUT4 to the plasma membrane. 

Perturbations of ER–mitochondria interactions either genetically or pharmacologically affect insulin action. For example, silencing of Grp75 or Mfn2 decreased insulin-stimulated phosphorylation of IRS2, Akt (PKB), and GSK3β [23]. In addition, pharmacological inhibition of IP3R also reduced insulin-stimulated Akt phosphorylation [23]. Akt, as an important signaling protein mediating the effect of insulin, was found to be recruited at the MAMs interface and phosphorylated on Ser473 in response to insulin. The in situ PLA technique confirmed interactions between Akt and IP3R1 as well as between p-Akt-IP3R1in hepatocytes [23]. Therefore, it seems that there are different pools of Akt in hepatocytes. MAMs-located Akt expression is altered under insulin resistance. Diabetic mice were characterized by increased phosphorylated Akt in MAMs [23]. Another MAMs-residing protein, mTORC2, was known to participate in mitochondrial metabolism, cell survival, and insulin signaling [33]. To be more specific, mTORC2 controlled insulin signaling by regulating Akt activation [33] and IRS-1 level [55]. Nitric oxide (NO) generated by eNOS has been shown to regulate the insulin signaling pathway in the liver. In addition, NO regulates mitochondrial fusion and mitochondrial biogenesis through the sGC/PKG (soluble guanylate cyclase/protein kinase G) pathway [56,57]. A recent study suggested that increasing NO concentration increased MAMs and improved insulin signaling through the sGC/PKG pathway, both in vivo and in vitro [58]. In addition, disrupting MAMs significantly blunted the effects of NO on MAMs and insulin response [58]. 

### 4.2. Ca^2+^ Homeostasis and Insulin Resistance

Ca^2+^ homeostasis is essential for cellular metabolism, and a variety of enzymes are activated upon elevation of intracellular [Ca^2+^]. The mitochondria and ER are two important organelles in Ca^2+^ regulation. In addition to playing distinct roles in cellular processes, the mitochondria and ER also interact physically and functionally to regulate Ca^2+^ homeostasis [59]. 

Mitochondrial dysfunction and chronic activation of ER stress (ERS) are essential components in the development of insulin resistance and diabetes. Mitochondrial dysfunction triggers ERS response, which increases cellular steady-state Ca^2+^ levels and facilitates p38 MAPK activation, thereby leading to hepatic insulin resistance and augmented gluconeogenesis [59]. A rise in the PC/PE ratio of ER inhibited SERCA function and Ca^2+^ transport activity, causing hepatic ERS and aberrant glucose metabolism. Conversely, overexpression of SERCA in the liver of *ob/ob* mice improved Ca^2+^ homeostasis and glucose tolerance [60].

The level of Ca^2+^ in different compartments of cells must be tightly regulated in order to maintain physiological homeostasis. When transport of Ca^2+^ from the ER to mitochondria is enhanced due to increased MAMs formation, mitochondrial Ca^2+^ overload and mitochondrial dysfunction are caused, as observed in the liver of obese mice [49]. To the contrary, knockdown of IP3R1 impaired Ca^2+^ release from the ER and profoundly improved insulin sensitivity in obese mice [49]. IP3R activity is also impacted by insulin signaling molecules. For example, Akt was reported to phosphorylate the IP3R channel, reducing its IP3-dependent Ca^2+^ release capacity [61]. As a mitochondrial enzyme that is abundantly expressed in the heart and skeletal muscle, PDK4 (pyruvate dehydrogenase kinase 4) is significantly upregulated during obesity and insulin resistance [62]. Recently, it was suggested that the role of PDK4 in insulin resistance was related to augmented MAMs formation, Ca^2+^ overload, and mitochondrial dysfunction [63]. To be more specific, PDK4 interacted with the IP3R1-Grp75-VDAC1 complex at MAMs. Obesity was found to be associated with increased PDK4 expression, enhanced MAMs formation, and insulin resistance, which could be ameliorated by PDK4 inhibition. Another MAMs-residing protein, promyelocytic leukemia (PML), is also likely to regulate insulin sensitivity by participating in Ca^2+^ homeostasis. Promyelocytic leukemia (PML) is a tumor suppressor that modulates apoptosis. PML is primarily enriched in a specific subnuclear structure called the PML nuclear body, while extranuclear PML is enriched at MAMs in the form of high-molecular-weight complexes with IP3R, Akt, and PP2A [64]. It was suggested that PML was crucial for Akt- and PP2A-dependent regulation of IP3R-mediated Ca^2+^ release from the ER [64]. In conclusion, Ca^2+^ dysregulation at MAMs is another mechanism accounting for insulin resistance [65]. Restorations of ER–mitochondria contacts and Ca^2+^ homeostasis represent a potential approach to correcting hepatic and muscle insulin resistance.

### 4.3. Metabolic Homeostasis and Insulin Resistance

Metabolic flexibility refers to the ability of organisms and cells to efficiently adapt metabolism to altered nutrient availability and requirements by substrate sensing, trafficking, storage, and utilization [66]. When organisms are unable to adapt their metabolism to energetic alterations, disrupted homeostasis results in metabolic disturbance, such as obesity, insulin resistance, and type 2 diabetes [47]. The mitochondria and ER are both organelles sensitive to cellular energetic levels. Mitochondria are involved in ATP synthesis, autophagy, and apoptosis, while the ER plays a key role in protein synthesis, folding, sorting, and delivery. 

There are multiple systems of quality control to ensure proper amounts and function of mitochondria, including mitochondrial biogenesis, mitochondrial fusion/fission, and mitophagy [67]. Upon starvation, Drp1 is phosphorylated, causing mitochondrial fusion, while mitochondria tend to maintain a fragmented state in nutrient excess [68]. In addition, acetylation of mitochondrial proteins and adjustment of mitochondria content are also key processes that guarantee metabolic flexibility [69,70]. Similarly, the ER triggers unfolded protein response (UPR) in the face of overload of glucose and fatty acid in order to restore metabolic homeostasis.

Nutritional state has been suggested to affect the amount and function of MAMs. PP2A is a serine/threonine phosphatase involved in energy metabolism. A significant reduction of MAMs amount was observed in the liver of fed mice compared with fasted mice, likely through the PP-PP2A pathway [51]. Moreover, the effect of nutritional state on MAMs was impaired in the liver of *ob/ob* and *CypD-KO* mice, implying that disruption of MAMs might result in mitochondrial dysfunction and hepatic insulin resistance [51]. As an MAMs-locating protein that forms a complex with Akt and PP2A [64], PML also participates in energy balance by regulating PPARs [71], FOXO1 [72], and AMPK [73]. Evidence showed that *PML-KO* mice exhibited augmented fatty acid metabolism, aberrant glucose metabolism, and insulin resistance in the skeletal muscle [73]. In addition, the activation of Akt was abolished with PML ablation, therefore causing AMPK activation [73].

**Figure 2:**
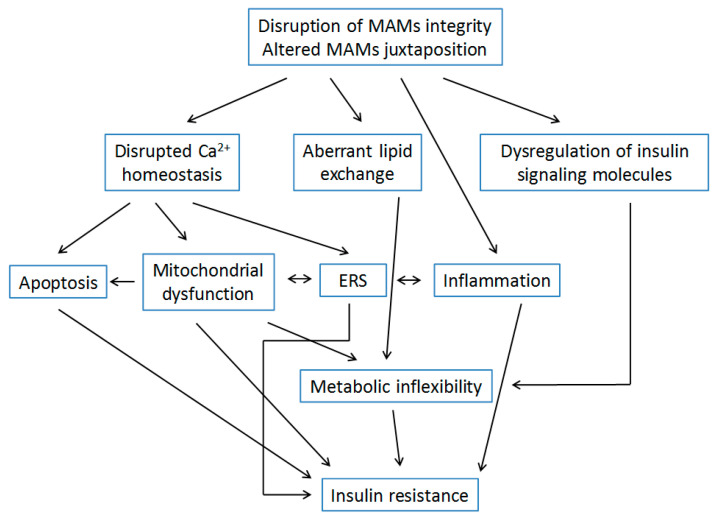
Schematic mechanisms by which alterations of the structure and functions of MAMs may contribute to insulin resistance.

Collectively, the research findings presented above support the close relationship between impaired MAMs functions and insulin resistance (Figure 2). Specifically, disrupted MAMs integrity and altered MAMs juxtaposition contribute to insulin resistance through impaired Ca^2+^ exchange, altered lipid metabolism, and dysregulated insulin signaling molecules. In addition, impaired Ca^2+^ homeostasis seems to be a crucial link between MAMs and the impaired signaling pathway, as modified apoptotic level, mitochondrial dysfunction, and excessive ERS are all associated with Ca^2+^ imbalance and insulin resistance.

## 5. MAMs, Insulin Resistance, and Exercise Intervention

It is well established that exercise deficiency contributes to the occurrence of obesity and type 2 diabetes (T2DM), which are both associated with systemic insulin resistance. On the other hand, solid evidence suggests that exercise plays an important role in the prevention and treatment of the above pathological conditions. Insulin sensitivity can be acutely improved by even a single bout of exercise. For example, acute one-legged exercise caused more rapid insulin-stimulated glucose uptake and glycogen synthase activity [74]. Similarly, a single day of moderate cycling exercise resulted in improved insulin sensitivity the next day [75]. Since both aerobic and resistance exercise could acutely improve insulin action, it is vital that insulin-resistant people exercise regularly to acquire continued health benefits [76]. Chronic aerobic exercise significantly lowered insulin concentration and the HOMA index in T2DM women [77]. Similarly, aerobic exercise training was proved effective in reducing fasting insulin level and the HOMA index in overweight/obese children/adolescents [78]. 

### 5.1. Exercise Intervention for Mitochondrial Quality Control and Insulin Resistance

For a long time, mitochondria were mainly considered as the site for energy production and oxidative phosphorylation. Now, we know that mitochondria are also indispensable for various cellular processes, including ROS production, autophagy, aging, apoptosis, redox signaling, etc. Dysfunction of mitochondria is associated with several diseases, including obesity, metabolic syndrome, cancer, and neurodegenerative diseases. Compromised mitochondrial activity and decreased mitochondrial content were reported in the skeletal muscle of adults with insulin resistance [79]. Decreased mitochondrial oxidative capacity causes over-accumulation of lipids, which, in turn, exacerbates insulin resistance. 

Mitophagy is a key process that influences mitochondrial quality, and is vital for maintaining insulin sensitivity. Studies investigating the role of mitophagy in insulin resistance have emerged over the last few years. As a mitophagy mediator, PTEN-induced putative kinase 1 (PINK1) expression was decreased under insulin resistance conditions. However, evidence from other studies showed that ablation of autophagy-related gene 7 (Atg7) or FUN14 domain-containing 1 (Fundc1) alleviated insulin resistance [80,81]. Therefore, it seems that a delicate level of mitophagy is required for maintaining insulin sensitivity. 

Exercise training poses positive effects on insulin sensitivity and glycemic control through mitochondrial adaptation. Training volume is a key factor in exercise-induced mitochondrial biogenesis [82]. Recently, researchers suggested that training intensity might be another important factor to stimulate mitochondrial function. For example, high-intensity interval training (HIIT) was superior to continuous aerobic training in improving insulin sensitivity and glucose tolerance and increasing muscle mitochondrial content [83]. In addition to training volume and intensity, exercise modality is another factor taken into consideration when prescribing an exercise regimen to overcome insulin resistance. For many years, strength training was known to influence muscle adaptation by increasing myofibrillar units [84]. However, recent studies suggested that resistance training could also elicit increased mitochondrial respiration to a similar extent to aerobic training in both lean subjects and T2DM patients [85,86]. Therefore, the combination of endurance and resistance training is considered a more effective behavioral intervention for T2DM according to the ADA/ACSM guidelines for diabetes. Like in muscle, exercise could also induce similar effects on insulin resistance and mitochondrial adaptation in the brain. For example, aerobic exercise improved HFD-induced mitochondrial dysfunction by ameliorating insulin resistance [87].

### 5.2. Exercise Intervention for ERS and Insulin Resistance

The ER is the main site in which folding and post-translational modifications of proteins occur. Under stressful conditions, the homeostasis of the ER is disrupted, and misfolded or unfolded proteins accumulate in the ER [88]. What happens next is that the UPR is triggered to restore ER function through three signal transducers, including activating transcription factor 6 (ATF6), inositol-requiring enzyme 1α (IRE1α), and protein kinase R-like ER protein kinase (PERK). When the UPR is not efficient enough to restore ER function, pathological changes are observed, including inflammation, insulin resistance, apoptosis, etc. [89]. Disruption of ER homeostasis is considered one of the prominent features of obesity, insulin resistance, and T2DM. Studies showed that aberrant activation of ERS is related to impaired insulin sensitivity. For example, animal models of insulin resistance, including HFD-induced obese mice and *ob/ob* mice, showed increased PERK phosphorylation [90]. 

Insulin resistance is usually associated with low-grade inflammation. Several studies have provided an intriguing link between inflammation, ERS, and insulin resistance. In particular, c-jun N-terminal kinase (JNK) and I kappa β kinase (IKKβ) are associated with ERS and act as inhibitors of insulin signaling, based on the fact that activation of IKKβ aggravates ERS and impaired insulin signaling [90,91]. Exercise training is believed to exert beneficial effects to correct ERS in most cases. For example, swimming training decreased pro-inflammatory molecules, such as JNK and NF-κB, reduced ERS, and improved insulin pathway in the adipose tissue and the liver of diet-induced obese rats [92]. Interestingly, concomitant intervention of HFD and exercise seemed to exert different effects on ERS and UPR compared to exercise intervention on the already obese rats [92,93]. When HFD and treadmill exercise began at the same time and both lasted for six weeks, exercise aggravated the UPR that was triggered by the HFD [93]. A possible explanation is that exercise induces adaptive changes to prepare for further potential challenges. It has been shown that aerobic exercise inhibited the activation of IKKβ and NF-κB and improved insulin resistance dependent on IL-6 and IL-10 in the hypothalamus [94]. In addition, exercise intensity seems to impact the effect of exercise intervention on ERS and insulin resistance. HIIT proved superior to low-intensity exercise in improving glycemic control, ERS, and glucagon-like peptide-1 (GLP-1) in T2DM adolescents [95]. Grp78, as the master regulator of the UPR, plays a vital role in modulating ERS. Under stressful events, ATF6, IRE1α, and PERK dissociate from Grp78 and are thus activated [96]. It is generally agreed that Grp78 is upregulated in obesity and insulin resistance, concomitant with increase of its targets, ATF6, IRE1α, and PERK [95,97]. However, the effect of exercise on Grp78 expression has not reached consensus. Some studies showed that exercise-induced improvement of insulin resistance was accompanied by an increase in serum Grp78 [95]. Moreover, HIIT seemed to induce a larger increase in Grp78 and better improvement in insulin sensitivity compared to low-intensity exercise in T2DM adolescents [95]. However, in other studies, supervised exercise training decreased the expression of Grp78 in both subcutaneous adipose tissue and plasma [97]. Therefore, the regulatory role of exercise for Grp78 and ERS is complex, and requires further investigations.

### 5.3. The Effect of Exercise Intervention on MAMs and Insulin Resistance

Studies investigating the impact of exercise intervention on MAMs have just emerged over the last few years. We now know that REDD1 (regulated in development and DNA damage responses 1)-mediated downregulation of protein synthesis following aerobic exercise was accompanied by a decrease in ER–mitochondria interaction [98]. In addition, swim training significantly lowered mitochondrial cholesterol level and enhanced Caveolin-1 expression in ALS mice, both of which were markers of MAMs [99]. However, the exact role of MAMs in cell adaptation to exercise stress is still unclear. Nevertheless, much evidence supported that exercise-induced alleviation of insulin resistance was achieved partly through MAMs components, and these findings will be reviewed in the following sections.

#### 5.3.1. Exercise, Ca^2+^ Homeostasis, and Insulin Resistance

Intracellular Ca^2+^ dynamics affect a variety of cellular functions, such as cell proliferation and differentiation, gene expressions, cellular signaling, etc. Studies demonstrate that Ca^2+^ transients and Ca^2+^ signaling are influenced by both acute and chronic exercises. For example, acute exhaustive exercise profoundly influenced Ca^2+^ signaling in lymphocytes [100]. Similarly, as an important signaling protein for glucose uptake following exercise, CaMKII was increased immediately after both acute aerobic and resistance exercise and returned to baseline after 3 h in the skeletal muscle of rats [101]. Three-week voluntary exercise also improved intracellular Ca^2+^ signaling in lymphocytes, and Ca^2+^-regulating genes such as IP3R2 and MCU were downregulated as adaptive changes [102]. 

Ca^2+^ regulation is one of the main functions of MAMs, and disruption of intracellular Ca^2+^ homeostasis is closely associated with insulin resistance. For example, HFD induced cardiac Ca^2+^ disturbance, insulin resistance, and compromised SERCA and RYR expressions, which were markedly improved by six-week treadmill exercise [103]. The alteration of Ca^2+^ homeostasis during insulin resistance is also related to fatty acid metabolism. To be more specific, fatty acid synthase (FAS) activity is increased in the skeletal muscle during diet-induced insulin resistance. The enhanced FAS activity facilitates synthesis of PE at the SR to maintain SERCA activity, which causes decreased cytosolic Ca^2+^, depressed Ca^2+^- and AMPK-dependent pathways, and muscle insulin resistance [104]. As the complex located at MAMs is in charge of Ca^2+^ transfer, the expression and function of IP3R-Grp75-VDAC1 are also affected by exercise. For example, the mRNA expression of IP3R2 was decreased in splenic lymphocytes by voluntary exercise, which was accompanied by improved intracellular Ca^2+^ signaling [102]. Grp75 is a family member of HSP70, and is actively involved in exercise-induced regulation of heat shock protein defense. The combination of exercise training and dietary intervention increased Grp75 in the skeletal muscle of individuals with impaired glucose tolerance [105]. The Grp75 mRNA level in a rat brain was also enhanced by exercise training [106]. However, the above studies only focused on the role of Grp75 as a heat stress protein, not as a Ca^2+^ signaling regulator. Therefore, further input is required to investigate the effect of exercise training on Ca^2+^ regulation related to MAMs.

#### 5.3.2. Exercise, MAMs Components, and Insulin Resistance

As a crucial tethering protein at MAMs, Mfn2 is actively involved in insulin resistance. Mfn2 in rat cardiac muscle was downregulated by HFD, and was upregulated by exercise training [103]. At the same time, lower mitochondrial ROS level, higher mitochondrial membrane potential, and attenuated insulin resistance were observed in HFD rats following exercise intervention, which confirmed the role of Mfn2 in exercise-induced improvement in insulin sensitivity [103]. In some cases, even though Mfn2 level was not changed in response to diabetes, the ratio of Mfn2 to Drp1 was decreased and was prevented by exercise [107]. Therefore, the balance between mitochondrial biogenesis and mitochondrial fission is shifted towards excessive fission under diabetic conditions, and could be partially corrected by exercise training.

Insulin resistance is associated with altered mitochondrial structure and impaired mitochondrial function. The dysfunctional mitochondria need to be eliminated by the mitophagic process [108]. During mitophagy, ER and mitochondria are detached from each other, promoting mitochondrial degradation. In the above process, PINK1 and Parkin mediate ubiquitination of an OMM protein such as Mfn2, and destroy the contacts between the mitochondria and ER [109]. Exercise training could induce autophagy in the normal skeletal muscle and lead to improvement in obesity-induced impairment of mitophagy [110,111]. Based on the above evidence, it is reasonable to propose that exercise training may relieve insulin resistance by upregulating Parkin to degrade Mfn2 and promoting detachment of the ER and mitochondria.

#### 5.3.3. Exercise, MAMs-Located Proteins, and Insulin Resistance

As an insulin-activated serine threonine kinase, Akt is actively involved in insulin- and exercise-induced glucose uptake. Even though some earlier studies showed that in vitro muscle contraction did not enhance Akt activity, it is generally agreed base on most evidence that the expression and activity of Akt are altered in response to exercise. In addition, the impaired Akt level in insulin resistance could be partially or fully reversed by exercise training. For example, eight-week swimming exercise profoundly enhanced the Akt and p-Akt^Ser473^ levels in the quadriceps muscle of HFD mice [112]. Similarly, six-week resistance training evidently increased p-Akt and GSK-3β in the skeletal muscle of diabetic rats [113]. In addition, voluntary exercise could improve insulin resistance in the liver of diabetic rats by inhibiting the iNOS level as well as the S-nitrosylation of Akt [114]. The impact of exercise on Akt modification is closely related to TRB3, a mammalian homolog of Drosophila tribbles that is actively involved in cell growth, cell differentiation, and metabolic processes [115]. Insulin resistance induced by HFD, obesity, and T2DM caused an increase in TRB3 [116], while exercise disrupted the interaction between TRB3 and Akt, therefore releasing Akt for further phosphorylation and activation [117].

Compared to the well-established role of mTORC2 in resistance-exercise-induced muscle hypertrophy [118], the physiological role of mTORC1 in response to exercise is less known. Studies have demonstrated that besides Akt, mTORC2 is also involved in insulin-induced glucose uptake. Moreover, *Rictor KO* mice exhibited impaired muscle glucose uptake during treadmill exercise, suggesting that mTORC2 was an essential component of exercise-induced glucose uptake [119]. In addition, HFD caused systemic insulin resistance and decreased muscle mTORC2 while combined interventions of dietary control and exercise training rescued the downregulated mTORC2 [120].

## 6. Conclusions

Since first observed in the 1950s, MAMs have been the focus of many scientific research groups. The significance of MAMs in insulin resistance has started to be elucidated, and growing evidence supports the involvement of this apposition in maintaining insulin sensitivity by mediating Ca^2+^ regulation, insulin signaling, and metabolic homeostasis. In addition, MAMs modulation is considered as a good target for potential pharmacological therapies and exercise interventions. However, even though ER–mitochondria tethers and MAMs-located molecules have been studied regarding their roles in regulating insulin sensitivity, the involvement of MAMs in glucose homeostasis is still not clear. Moreover, some conflicting results were drawn due to the different disease models (obesity, type 2 diabetes, insulin resistance, NAFLD) and intervention methods (pharmacologic vs. genetic manipulation) used. Therefore, the role of ER–mitochondria contacts in the pathogenesis of insulin resistance is still to be explored. Furthermore, the impact of exercise intervention on MAMs modification remains an open question. Much input is required to understand whether and how alterations of MAMs’ structure and functions are involved in insulin sensitivity enhancement by different types of exercise.

## Abbreviations

ER	Endoplasmic reticulum
MAMs	Mitochondria-associated ER membranes
NAFLD	nonalcoholic fatty liver disease
PACS-2	phosphofurin acidic cluster sorting protein 2
Mfn2	mitofusin 2
IP3R	inositol 1, 4, 5-triphosphate receptor
VAPB	vesicle-associated membrane-protein-associated protein B
PTPIP51	protein tyrosine phosphatase-interacting protein 51
CNX	calnexin
SERCA	sarco/ER Ca^2+^ ATPase
Sig1R	sigma-1
Ero1α	endoplasmic reticulum oxidoreductin-1α
PSS	phosphatidylserine synthase
CypD	cyclophilin D
mTORC2	mammalian TOR complex 2
OMM	outer mitochondrial membrane
TpMs	trichoplein/mitostatin
mPTP	mitochondrial permeability transition pore
PE	phosphatidylethanolamine
PC	phosphatidylcholine
PMCA	plasma membrane Ca^2+^ ATPases
RyR	ryanodine receptor
MCU	mitochondrial Ca^2+^ uniporter
PC2	polycystin 2
PLA	proximity ligation assay
HFD	high-fat diet
Giamp5	GTPase of immune-associated protein 5
IRSs	insulin receptor substrates
sGC/PKG	soluble guanylate cyclase/protein kinase G
ERS	ER stress
PDK4	pyruvate dehydrogenase kinase 4
PML	promyelocytic leukemia
UPR	unfolded protein response
T2DM	type 2 diabetes
PINK1	PTEN-induced putative kinase 1
Atg7	autophagy related gene 7
Fundc1	FUN14 domain-containing 1
HIIT	high-intensity interval training
ATF6	activating transcription factor 6
IRE1α	inositol-requiring enzyme 1α
PERK	protein kinase R-like ER protein kinase
JNK	c-jun N-terminal kinase
IKKβ	I kappa β kinase
GLP-1	glucagon-like peptide-1
REDD1	regulated in development and DNA damage responses 1
FAS	fatty acid synthase
